# A review on antimicrobial peptides databases and the computational tools

**DOI:** 10.1093/database/baac011

**Published:** 2022-03-19

**Authors:** Shahin Ramazi, Neda Mohammadi, Abdollah Allahverdi, Elham Khalili, Parviz Abdolmaleki

**Affiliations:** Department of Biophysics, Faculty of Biological Sciences, Tarbiat Modares University, Jalal Ale Ahmad Highway, Tehran 14115-111, Iran; Department of Medical Biotechnology, Faculty of Allied Medical Sciences, Iran University of Medical Sciences, Hemmat Highway, Tehran 1449614535, Iran; Institute of Pharmacology and Toxicology, University of Bonn, Biomedical Center, Venusberg Campus 1, Bonn 53127, Germany; Department of Biophysics, Faculty of Biological Sciences, Tarbiat Modares University, Jalal Ale Ahmad Highway, Tehran 14115-111, Iran; Department of Plant Biology, Faculty of Biological Sciences, Tarbiat Modares University, Jalal Ale Ahmad Highway, Tehran 14115-111, Iran; Department of Biophysics, Faculty of Biological Sciences, Tarbiat Modares University, Jalal Ale Ahmad Highway, Tehran 14115-111, Iran

## Abstract

Antimicrobial Peptides (AMPs) have been considered as potential alternatives for infection therapeutics since antibiotic resistance has been raised as a global problem. The AMPs are a group of natural peptides that play a crucial role in the immune system in various organisms AMPs have features such as a short length and efficiency against microbes. Importantly, they have represented low toxicity in mammals which makes them potential candidates for peptide-based drugs. Nevertheless, the discovery of AMPs is accompanied by several issues which are associated with labour-intensive and time-consuming wet-lab experiments. During the last decades, numerous studies have been conducted on the investigation of AMPs, either natural or synthetic type, and relevant data are recently available in many databases. Through the advancement of computational methods, a great number of AMP data are obtained from publicly accessible databanks, which are valuable resources for mining patterns to design new models for AMP prediction. However, due to the current flaws in assessing computational methods, more interrogations are warranted for accurate evaluation/analysis. Considering the diversity of AMPs and newly reported ones, an improvement in Machine Learning algorithms are crucial. In this review, we aim to provide valuable information about different types of AMPs, their mechanism of action and a landscape of current databases and computational tools as resources to collect AMPs and beneficial tools for the prediction and design of a computational model for new active AMPs.

## Introduction

### Antimicrobial peptides (AMP_S_)

In the past few decades, antibiotics have been used to defeat infectious diseases and most of them were discovered during the 1940s to 1960s (1). However, the increased usage of conventional antibiotics has resulted in significant rates of resistance in microorganisms, raising concerns about the spread of infectious diseases. Antimicrobial resistance (AMR) could be detected using a variety of experimental and technological methods, such as phenotypic and molecular-based techniques, as well as the more recently developed sequencing whole-genome sequencing (WGS) and whole-genome metasequencing (WGM), MALDI-TOF MS and Infrared (IR) spectroscopy (2). AMR has become an increasingly urgent challenge in healthcare (3), with antimicrobial-resistant infections estimated to increase to 10 million cases annually by 2050 (4). It has recently been estimated that at least 700 000 people die from antimicrobial-resistant (AMR) infections every year (5, 6). In light of the 2020 COVID-19 pandemic (4, 7), antibiotic-resistant issues are even more exacerbated due to increased different antibiotics prescribed to COVID-19 patients (8). In addition, the existing repertoire of antibiotics does not offer solutions for multidrug-resistant bacteria, so-called superbugs. Therefore, the global health burden has led to an urgent demand for the expansion of new classes of antibiotics. A highly promising approach to overcome this problem is the development of AMP-based drugs.

AMPs are naturally present in the innate immune system and have broad-spectrum antimicrobial properties aiding in the defense against invading microorganisms (9). They are short cationic peptides of up to 100 amino acids (9), with an alpha-helical secondary structure and amphiphilic surface properties, which are considered essential for establishing antimicrobial activity (10). AMPs’ main mechanism of action (MOA) is the disruption of the target microorganism’s cell membrane, through hydrophobic or electrostatic interactions, causing lysis of the cell (11). AMPs pose several advantages over conventional antibiotics including the rapid killing of bacteria owing to their broad-spectrum activity, antimicrobial immunomodulatory effects and the less likelihood of AMR (11, 12).

The initial study on AMPs was performed by Dubos *et al.* in 1939 over the soil bacillus strain named gramicidin which is suitable for the typical treatment of wounds and ulcers (13). Recently, AMPs have drawn much attention due to their biological and biomedical applications especially in designing various types of APM-based drugs (14, 15). Over 5000 AMPs have been so far identified or synthesized in a wide variety of organisms ranging from prokaryotes (e.g. archaea and bacteria) to eukaryotes [e.g. yeasts, fungi, viruses, parasites, protozoa, insects, plants and animals (invertebrates and vertebrates)] (13). For example, more than 300 different AMPs exist in the skin of frogs, which is a crucial part of the innate immunity against a wide range of microbes including viruses, bacteria and fungi (13). The gene expression of AMPs is correlated with their activity and maintained at an optimal level. While some AMPs have tissue-specific expression patterns like human β-defensin 1 (hBD-1) or mouse β-defensin 1 (mBD-1), their dysregulations are attributed to the pathological state (16). It has been shown that β- defensins are upregulated in pneumonia (17) and cystic fibrosis (18),. while the expression of hBD-2 and hBD-3 is decreased in atopic dermatitis (19).

Besides their antimicrobial activity and immune regulatory roles, AMPs have antiparasitic, antiviral, anti-biofilm, anti-inflammatory, anticancer, insecticidal, wound-healing and/or chemotactic properties which make them interesting candidates for novel therapeutic strategies (20–22). Therefore, AMPs are capable of targeting different types of diseases such as infectious diseases, diabetes, cancer, cardiovascular disease and Alzheimer’s disease (23–25). Although the antimicrobial mechanisms of AMPs remain poorly understood, it has been known that AMPs act through the destruction of cell membranes, interference with DNA, RNA, disruption of enzymatic/protein activity, interference with cell division and the inhibition of cell wall synthesis (26). In particular, buforin II is a histone-derived AMP that is mainly found in frogs that destroy Escherichia coli (E.coli) by binding their DNA and RNA, but without bacteria membrane permeabilization. In addition, it has been shown that β defensin 4, α defensin 1 and PR-39 play key roles by targeting the intracellular bacterial components in humans (27). Nisin and lysozyme as encouraging examples of AMPs which are firstly isolated from Lactococcus lactis subsp, human tissues and body fluids, respectively (28). Later, a great number of membrane-lytic peptides were extracted from amphibians, insects and mammals in the 1980s (24). For instance, melittin, mastoparans, cecropins, defensins and magainins are isolated from bee wasp venom, insects, mammalian neutrophils and frog skin, respectively (29). Since then, compelling evidence has demonstrated that there is an interspecies variation in either the sequence or the structure of AMPs isolated from different organisms (30).

Despite the capacity of AMPs as promising alternatives to conventional antibiotics, the number of issues related to the production of AMPs has limited AMPs’ applications in clinics (13). These difficulties are assigned to the high toxicity, reduced activity due to the extreme environmental conditions (susceptibility to proteases and extreme pH), lack of specificity, folding problems in large AMPs, bacterial resistance and highly expensive production costs (13). In general, AMPs are short in length, highly selective, efficacious and generally well-tolerated (31–33). Nevertheless, large-scale detection of AMPs is costly and challenging. In current years, computational methods have attracted considerable attention to AMP prediction (34). To resolve this issue, many computational methods have been recently developed to predict and design putative AMPs *in silico*; databases and computational methods as common tools for the prediction of AMPs contain a great number of AMPs. The Antimicrobial Peptide Database (APD3), is the commonly used AMP database, which covers more than 2600 AMPs (35). It is difficult to classify natural AMPs due to their diversity (36). AMPs are particularly categorized based on their source, activity, structure, sequence, biosynthesis or functions (20, 21, 37). In the following section, we provide a scheme for both the structure and function of AMPs.

### Functions, structure and major activities of AMPs

AMPs are diverse and distinct molecules that are distinguished by their chemical structures and amino acid composition. Most AMPs are less than 50 amino acids, with net cationic charge ranging from +2 to +9 and amphiphilic with the molecular weight of <10 kDa containing hydrophobic residues (34, 38). On the other hand, most of these cationic peptides are considered as a heterogeneous group with a length between 12 and 48 residues of amino acid and hydrophobic characteristics to form amphipathic-helix in solvents as fluoro-ethanol that mimics cell membrane (39). Notably, a direct correlation between the charge of AMPs and their antimicrobial activities has been indicated; an increase in the charge of peptides leads to an improvement in the activity of peptides. As an illustration, an increase in the charge of magainin 2 from +3 to +5 enhances the antibacterial activity against both Gram-positive and Gram-negative bacteria. In vice versa, these AMPs do not adversely affect eukaryotic membranes. For example, an increase to +6 or +7 leads to an increase in hemolytic activity and the loss of antimicrobial activity (37).

All AMPs were considered cationic in the late 90s, but later with the discovery of negatively charged AMPs in 1997, this view was changed (13). Some natural peptides are negatively charged, such as maximin H5, dermcidin and enkelytin, which are enhanced by their activities by combination with zinc or highly cationic peptides (13, 40). AMPs are phospholipid-rich and could act through a lipid bilayer in a detergent-like manner, solubilizing it into micelles and/or allowing it to penetrate by forming pores. Both interactions generate transient membrane permeation and cytoplasmic leakage depending on the AMP concentration which might cause cell death (41). In addition, AMPs interfere with the intracellular activities of the bacteria by the inhibition of intracellular activities, such as cell division and biosynthesis of proteins, nucleic acids and components of the cell wall (41).

AMPs with the structural and functional variety are obtained from three sources: natural sources like microbes, plants, animals and insects, which are synthesized by ribosomal or nonribosomal approach, recombinantly expressed in microorganisms and chemically synthesized sources (42). While ribosomal AMPs are produced by all the species of life such as mammals, birds, amphibians, insects, plants or particular microorganisms, nonribosomal AMPs are mainly synthesized by bacteria and fungi (43, 44). In terms of chemically synthesized peptides, firstly Bruce Merrifield introduced solid-phase peptide synthesis (SPPS) in 1963 (45). Currently, the chemical synthesis of peptides has been significantly developed owing to the reasonable production price compared to recombinant production (46). Chemically synthesized AMPs are advantageous in comparison to other methods since there is a possibility to produce unnatural amino acids, D-amino acids and other building blocks with a noticeable quantity and quality (47).

**Figure 1.**
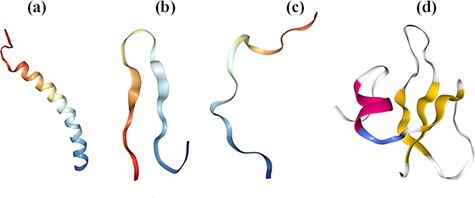
Structural diversity of AMPs based on their secondary conformations using Protein Data Bank (PDB). [(a) **2K6O:** Antimicrobial Peptide, the α-helical structure of cathelicidin LL-37 in *Homo sapiens*. (b) **1RKK:** Antimicrobial Peptide, β-sheeted polyphemusin in *Limulus polyphemus*. (c) **1G89:** Antimicrobial Peptide, extended indolicidin in Bos taurus. (d) **1FQQ:** Antimicrobial Peptide, Antibiotic and mixed structures like human β- defensin-2 in *Homo sapiens*].

A broad-spectrum function and the structure of APMs are excessively attributed to the post-translational modifications (PTMs) mediated by proteolytic cleavage, phosphorylation, glycosylation, amidation, halogenation, D-amino acids, disulfide bridge and cyclization (13, 48). The 3D structures of AMPs have been determined by circular dichroism spectroscopy, X-ray crystallography and nuclear magnetic resonance (NMR) (28, 49). The first structure human α-defensin and neutrophil peptide 3 was characterized by X-ray crystallography in 1991, and then the structure of the human neutrophil peptide 1 was determined by NMR (50, 51). AMPs are classified based on their structure into four broad families: α-helix, β-sheet, loop and extended. AMPs with α-helix and β-sheet structures are the most prevalent structures in nature (Figure 1) (13). Cathelicidin LL-37, human lactoferricin, magainin and cecropin are the most studied α-helical peptides (52–55). Studies showed that helical peptides are destabilized in an aqueous solution and undergo an amphipathic structure upon interaction with the biological membrane (56). Cathelicidin contains 12–80 amino acids and adopts a diversity of structures and exists in a large group of mammals such as mice, goats, sheep, horses and bovines. Lactoferrin is found in neutrophils and the secretions of the exocrine glands of mammals. Magainins are a class of helical peptides that are mainly effective against Gram-positive and Gram-negative bacteria, fungi, yeast and viruses, and isolated from the African clawed frog *Xenopus laevis* (57). Cecropins are the first discovered AMPs in eukaryotes in the silk moth which have cationic, amphipathic activities against Gram-positive and Gram-negative bacteria and fungi. In recent years, these peptides have been identified in fruit flies (Drosophila) and marine invertebrates such as shrimp, oysters and horseshoe crabs (37). Aurein peptides are another example of α- helical AMPs and consist of more than 30 aurein peptides and five different families primarily secreted from the granular dorsal glands of the Australian Green and Golden Bell Frog *Litoria aurea* and the Southern Bell Frog *Litoria*  *raniformis* (58). Most Aurein peptides are active against Gram-positive bacteria, such as *Staphylococcus aureus* and *Staphylococcus epidermidis*. Furthermore, aurein peptides 1.2, 3.2 and 3.3 show the greatest activity against more than 30 various types of cancer (37). Interestingly, Aurein peptides are rich in specific amino acids. For instance, histatin as an antimicrobial peptide isolated from human saliva is histidine-rich and defeats *Candida*  *albicans* mushrooms (59, 60) while bactenecin Bac-5 and Bac-7 peptides are prolin-rich and possess an irregular structure (61). β-sheet peptides contain cysteine residues with the rigid structure stabilized with disulfide bonds and an unaltered conformation in contact with the cell membrane. Protegrins (a member of the cathelicidin family), defensins and tachyplesins have the β-sheet structure (37). AMPs can be found in leaves, flowers, seeds and tubers of plants. Some are cysteine-rich with multiple disulfide bonds playing key roles in high chemical, thermal and proteolytic stability. Defensins, thionins, hevein-like peptides, knottin-type peptides (linear and cyclic), α-hairpinins, lipid transfer proteins and the snakins family are the examples of plant-derived AMPs (62, 63).

### Mechanism of AMPs action

**Figure 2.**
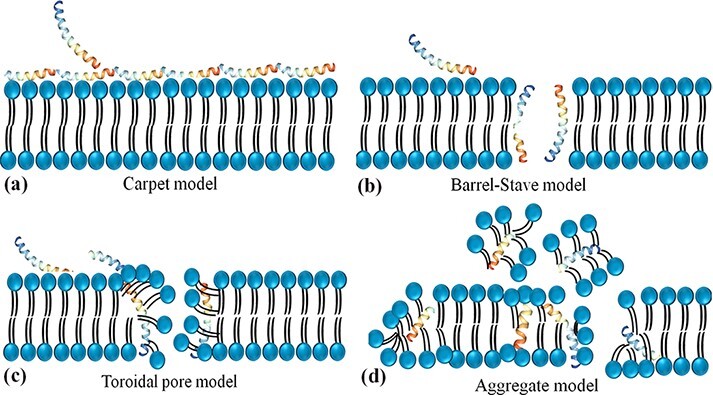
Schematic representation of the potential mechanism of membrane disruption and/or translocation by antimicrobial peptides. **(a) Carpet model**: Another face of the membrane is covered by AMPs to form a ‘carpet’ and the membrane undergoes some perturbation and deformation. **(b) Barrel-Stave model:** AMPs interact laterally and form transmembrane pores. **(c) Toroidal pore model:** AMPs penetrate the bilayer membrane and form a toroid of high curvature. **(d) Aggregate model.**

Antimicrobial peptides are unique molecules and their MOA has been studied extensively since they were discovered. It is important to understand the MOA of these AMPs to facilitate further development as therapeutic agents. It was originally thought that membrane targeting was the only MOA, but there is increasing evidence now that AMPs have other modes of action. The MOA can be divided into two major classes: direct killing and immune modulation (64). As described above, AMPs have immune-modulatory and antimicrobial roles through directly targeting membrane and non-membrane regions of microbes (27). In most cases, the positive net charge of AMPs displays a significant role in antimicrobial activity via the strong interaction with negatively charged bacteria surfaces and thus disruption of the physical integrity (26). Four different models for the antimicrobial activity of AMPs have been suggested which lead to membrane disruption through permeabilization, including a barrel-stave, aggregate channel or toroidal pore and carpet models (Figure 2) (65).

In the barrel-stave model, AMPs are inserted perpendicularly in the membrane bilayer and form a pore. In this pore, the hydrophobic sides interact with the lipids of the membrane and form the interior side of the channel. In this way, they act as pore formers or act as metabolic inhibitors in bacteria (66). Compelling evidence showed that alamethicin (67), pardaxin (68) and protegrins (66) form barrel-stave channels. In the toroidal pore model, the peptides are inserted vertically into the membrane and form a curve structure and a pore through the peptides and the head phosphates of phospholipids. Some peptides are permitted to enter the cytoplasm using this model and target intracellular components (69) including magainin 2 (70), lacticin Q (70), aurein 2.2 (71) and melittin (70). In the carpet model, the AMPs cover the surface of the membrane and cause tension which leads to membrane disintegration and micelle formation. Some AMPs such as cecropin (72), indolicidin (73), aurein 1.2 (73) and LL-37 (74) form carpet models. These three models are suggested that lead to the breakdown of membrane integrity resulting in membrane dysfunction, and leakage of metabolites and ions (75). This membrane permeabilization is also contributed to the subsequent translocation of AMPs into the intracellular region and blocks critical cellular processes such as protein/ nucleic acid synthesis, enzymatic/protein activity, protein folding, intracellular pathways and/or cell wall synthesis (75).

Furthermore, AMPs are mainly produced by some immune cells such as neutrophils and macrophages and exert immunomodulatory activities such as the recruitment and activation of immune cells, initiation of adaptive immunity, reduction of inflammation (27), chemo attraction of immune cells, induction of chemokine, cytokine, and histamine production and secretion, wound healing stimulation, angiogenesis and adjuvant city (76).

The rest of the paper is structured as follows. The five most studied AMPs are described in the section ‘Classification of AMPs based on biological functions’. In the next section, major AMP databases will be reviewed. Afterward, computational methods for the prediction of AMPs will be described in detail. Finally, available tools for AMP prediction will be reviewed in the section ‘Tools for AMP prediction’.

## Classification of AMPs based on biological functions

3000 synthetic and natural AMPs have been identified and 7 have received approval from the U.S. Food and Drug Administration (FDA) (77). In humans, AMPs are mostly present in lymphocytes and epithelial surfaces of different organs including the eye, skin, lung, intestines, etc (22). A great number of AMPs (e.g. alpha-defensins, lysozyme, etc) are produced in paneth cells, primary secretory epithelial cells in the small intestine, thereby, controlling the number of bacteria in the small intestine. Defensins, lysozyme and cathelicidins in the tear fluid protect the eyes from infections (22).

**Figure 3.**
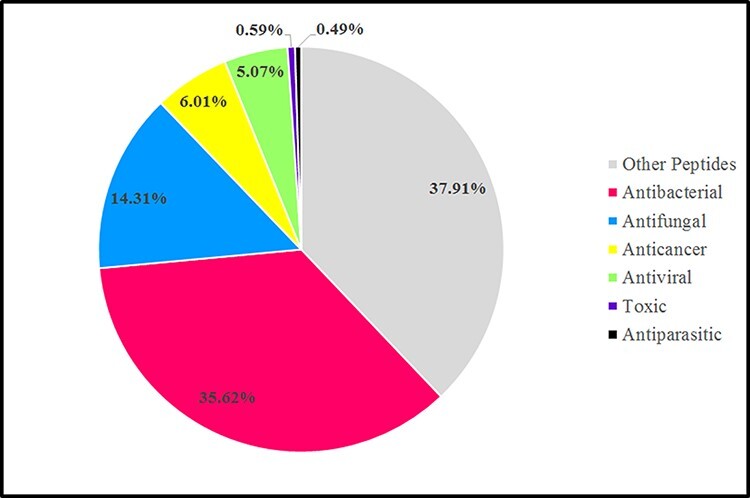
Graphic representation sources of AMPs in the antimicrobial peptide database (dbAMP 2.0) in 2022 (78).

According to AMPs’ biological functions, AMPs could be divided into various groups such as antibacterial peptides (ABPs), antiviral peptides (AVPs), antifungal peptides (AFPs), anticancer peptides (ACPs) and antiparasitic peptides (APPs). Categories of AMPs discussed based on the Databank antimicrobial peptides (dbAMP). In the dbAMP database, ABPs comprised the largest proportion, approximately 35.62%, followed by AFPs, which account for 14.31%, and, ACPs, AVPs, toxic peptides and APPs account for about 6.01%, 5.07%, 0.59% and 0.49%, respectively. In addition, the dbAMP database contains 37.91% of various other peptides, which are known as disease-associated peptides and new mechanism-associated peptides (Figure 3) (78).

### Antibacterial peptides (ABPs)

ABPs are cationic AMPs, which have been well documented for their role in the development of antibacterial drugs. Despite eukaryotic membranes with zwitterionic lipids, bacterial membrane lipids such as phosphatidylglycerol (PG), cardiolipin (CL) or phosphatidylserine (PS) have negatively charged residues with lipids bearing phospholipid head groups which predispose their membrane to the disruption (34). These AMPs frequently fold into amphiphilic α helices exposing both hydrophobic and hydrophilic surfaces (79). Nisin (as an ABP) and vancomycin (as an antibiotic) function through the blockage of cell wall synthesis (13). They are accumulated on the negatively charged outer membrane of the Gram-negative bacteria or the cell wall of the Gram-positive bacteria leading to the formation of membrane-spanning pores, inhibition of cell wall biosynthesis, and thereby, disruption of membrane integrity (80). Once the bacteria are penetrated, they interact with intracellular components to destroy them. Some of ABPs with low concentrations and without interacting with the membrane, lead to the death of bacteria by inhibiting many significant pathways inside the cell such as DNA replication and protein syntheses such as buforin II, drosocin, pyrrhocoricin and apidaecin (13).

The primary link of peptides with the bacterial membrane happens via electrostatic interactions between the cationic peptide and anionic lipopolysaccharides (LPS) in the outer membrane leading to membrane disorder (49). Bacteriocins are the subset of ABPs which are mainly classified into two categories: lantibiotics and non-lantibiotics. Lantibiotics comprise the nonnatural amino acid lanthionine (37). Nisin and mersacidin belong to the family of antibiotics which are produced by *Lactococcus lactis* and Bacillus sp, respectively. It has been shown that nisin and mersacidin are active against antibiotic-resistant Gram-positive bacteria (37).

### Antiviral peptides (AVPs)

Viral diseases are the foremost cause of illness and mortality worldwide and more than 200 viruses are accounted for a variety of human diseases (81) like influenza (IAV), West Nile Virus (WNV), Epstein-Barr virus (EBV), cytomegalovirus (CMV), respiratory syncytial virus (RSV), hepatitis B and C viruses (HBV and HCV, respectively), herpes simplex virus (HSV), human immunodeficiency virus (HIV), rabies virus and Ebola virus (81–83). So far, 1.5 million deaths due to HIV, 400 million cases due to HBV or HCV, 80% of liver cancer deaths related to hepatitis viruses, 500 000 cervical cancer cases, and 250 000 deaths associated with HPV have been reported by the World Health Organization (WHO) (82). Among over 60 antiviral drugs approved by the FDA, around half of them have been used to treat HIV-1 and the rest are used to treat HBV, HSV, varicella-zoster virus (VZV), CMV, IAV and HCV (83). Therefore, the low access to therapeutic possibilities for many viral infections emphasizes the efforts to develop new and more effective antiviral drugs. In recent years, 15 peptide-based drugs which are in different stages of clinical trials have provided new opportunities to combat wide-spectrum viruses. However, none of the ACPs has cationic properties and are widely used (13, 81). AVPs have cationic features with virucidal activity: They mainly interfere with the virus replication by targeting DNA or RNA after binding to their envelopes and causing membrane instability (13, 81). As a result, antiviral drugs have mostly two types of mechanisms of action, i.e. virus targeting and host targeting drugs that can inhibit various transcriptional and replication-related enzymes and lead to destroy a viral pathogen or inactivate their infectiveness (13).

### Antifungal peptides (AFPs)

Fungi are considered as a unique kingdom with different characteristics than other eukaryotic kingdoms such as plants, animals and are more complex than viruses or bacteria. They are used in the industry to produce peptides, vitamins, antibiotics, organic acids, enzymes, etc. (84). The cell wall of fungi is composed of chitin, 1,3-β- and 1,6-β- glucans, proteins and other polymers of the complex cellular organizations (85). Over 400 species are accountable for various infectious diseases in humans (86). In most cases, fungal infections may lead to serious problems in people who are very sensitive, such as mainly immunocompromised, elderly, and transplanted subjects, cancer patients and premature infants, and the elderly with significant associated morbidity/mortality (85, 87). Recent reports suggest that current antifungal drugs have caused a significant rise in drug-resistant strains and their incidence is on the increase. Therefore, is need alternative antifungal drugs that are capable of overcoming resistance mechanisms (88, 89).

AFPs have provided a great extent of advantages by being effective against multiple targets and developing less resistance (88, 89). AFPs have been extracted from many natural resources such as plants (85), amphibians (90), bacteria (91), fungi (92), marines (93) and insects (94). Most AFPs have a length of ∼50 amino acid residues in linear or cyclic structures with hydrophobic or amphipathic properties, cationic and cysteine-rich proteins (CRPs) (95, 96). AFPs play key roles in many action mechanisms of cells such as inhibition of DNA, RNA and protein synthesis, binding to DNA or RNA, membrane permeabilization, inhibition of cell wall synthesis and enzyme activity, induction of apoptosis and repression of protein folding (95). Lytic peptides of fungi bind to the membrane surface and can destroy the cell membrane with or without crossing the membrane (13). There is no direct correlation between the structure of AFPs and the type of target cell. For example, AFPs have members from various structure classes like α-helical (D-V13K and P18), extended (indolicidin) and β-sheet (defensins) (13).

### Anticancer peptides (ACPs)

Despite a wide variety of cancer treatment methods, this disease is one of the most common causes of death worldwide (97). A common method for treating cancer is chemotherapy, which damages both cancerous and normal cells by inhibiting DNA replication. On the other hand, chemotherapy drugs cause chemical resistance, which results in a low success rate and an increased risk of recurrence (98, 99). Besides, there is a well-known reciprocal relationship between infection and cancer which is associated with the weak immune system to provide a proper situation for cancer and infection (100–102). In recent years, some AMPs have exhibited antitumor activity called anticancer peptides (ACPs), acting as mitogens and signaling molecules. ACPs are described as promising chemotherapeutic drugs in the future, particularly owing to the low resistance, minimal side effects, high specificity and proper solubility (103). ACPs are functionally categorized into two classes: ACPs with dual activity against cancer cells and bacteria, but not normal cells, and ACPs with cytotoxic function against microbial infections, cancer cells and also normal cells (104, 105). These peptides are typically less than 50 amino acids and possess high hydrophobicity and positive net charge (106).

The physicochemical properties of cancer cells provide the basis for the function of ACPs. Generally, eukaryotic cells are bilayered membranes containing asymmetric zwitterionic phospholipid composition (107). The double-layer membrane has phosphatidylcholine (PC) and sphingomyelin (SM), phosphatidylethanolamine (PE) and phosphatidylserine (PS) (107). Despite the healthy cells, PS, a phospholipid with a negative net charge, is translocated from the inner to the outer membrane in cancer cells (108). Owing to the highly cationic and amphipathic features, AMPs target cancer cells exist. Thus, ACPs are attached according to their cationic and amphipathic characteristics by electrostatic interactions with a negative net charge in the outer membrane of cancer cells. These anionic molecules can affect with the utmost selectivity and toxicity through the destabilization of the membrane integrity (108). In addition to the membranolytic mechanism, ACPs promote necrosis or apoptosis in cancer cells by inducing mitochondria-derived pathways (105, 108–110). Hence, the negative charge of the cancer cell membrane is an important factor to promote the ACPs’ electrostatic interaction (111).

### Antiparasitic peptides (APPs)

Parasitic diseases like malaria, leishmaniasis, trypanosomiasis, schistosomiasis and chagas have imposed a great burden on humans, by mostly affecting the poor population. The lack of suitable vaccines and drugs without causing resistance necessitates the development of new drugs (13). APPs are short in length (∼ 5–30 amino acids) which target Protozoa through plasma membrane disruption and consist of a smaller group of AMPs compared to the other four AMP classes (13). Magainins and cecropins are the first APPs, reported 20 years ago, which are active against *Paramecium caudatum* (13). APPs can be isolated from the host including mosquitoes and other invertebrates (112–115). It has been reported that APPs have great potential for treating diseases including protozoan parasites (114). Despite the multicellularity of some parasitic microorganisms, antiparasitic peptides act in the same way as other AMPs, directly targeting and killing cells by destabilizing cell membranes (13).

## Major AMPs databases

A multitude of evidence has shown that AMPs have remarkable antimicrobial effects, particularly against the increasing number of resistant microbes. However, many of them are not approved by FDA and fail before or during clinical trials (37). To meet this need, several databases have provided more classified information for the effective design and construction of AMPs. Databases enable users to search and mine extensive information on the peptide structure, chemical modifications, bioactivities and classification. Tables 1 and 2 present a list of databases. The AMPs databases are classified into two main groups: general databases and specific databases. The general databases contain the whole types of AMPs irrespective of a given peptide family while specific databases cover information related to a certain class of AMPs (e.g. only defensins or cyclotides) or hold a supergroup of AMPs (e.g. only plant peptides or only cyclic peptides). Currently, there is not a universal database with all AMP data, the information is divided into several databases (Tables 1 and 2), and there exists an overlap as well between AMP databases; nonetheless, each database contains some exclusive sequences (63).

### General AMPs databases

In the subsequent section, the six comprehensive general databases are defined briefly. In addition, Table 1 reviews the current main public AMPs general databases.

**Table 1.** A description of existing antimicrobial general databases

		General Statistics				
Database Name	Number of covered classes and AMPs	Size	Type of Database^a^	Type of Data	Years	URL
dbAMP	26 major Functional activity classes in 3044 organism	∼26 440	Exp. and Pred. Secondary	Natural and Synthetic	2022	http://awi.cuhk.edu.cn/dbAMP
DBAASP	Antibacterial, antifungal, antiviral, anticancer, and antitumor in seven organisms and cancer cells and mammalian cells	∼15 700	Exp. and Pred. Secondary	Natural, synthetic, and patent	2021	http://dbaasp.org/home
LAMP	8 major functional classes and 38 functional activities	∼ 23 250	Exp. and Pred. Secondary	Natural, synthetic, and patent	2020	http://biotechlab.fudan.edu.cn/database/lamp/index.php
DRAMP	Antimicrobial, antifungal, antiviral, anticancer, antitumor, antiprotozoal, and insecticidal	∼ 22 250	Exp. and Pred. Secondary	Natural, synthetic, patent, and AMPs in drug development	2019	http://dramp.cpu-bioinfor.org/
dbAMP	26 major Functional activity classes in 3044 organism	∼26 440	Exp. and Pred. Secondary	Natural and Synthetic	2018	http://csb.cse.yzu.edu.tw/dbAMP/
InverPep	Invertebrates *phyla Arthropoda, Mollusca, Nematoda, Annelida, Echinodermata, Platyhelminthes, Placozoa*, the Hydridae family (*Cnidaria*) and the subphylum Tunicata (*Chordate*)	∼770	Exp. Primary	Natural	2017	http://ciencias.medellin.unal.edu.co/gruposdeinvestigacion/prospeccionydisenobiomoleculas/InverPep/public/home_en
CAMP	Antibacterial, antifungal and/or antiviral	∼8160 sequences, and 757 structures	Exp. and Pred. Secondary	Natural Predicted and patented	2016	http://www.camp3.bicnirrh.res.in/
MEGARes	Antimicrobial compounds, e.g. drugs, biocides, multi-compound and metals	∼8000	Exp. Primary	Natural	2016	http://megares.meglab.org/
ADAM	archaea, bacteria, plants and animals	∼7000	Exp. Primary	Natural	2015	http://bioinformatics.cs.ntou.edu.tw/adam/index.html
APD	Antibacterial	∼1230	Exp. and Pred. Primary	Natural and patent	2008	https://webs.iiitd.edu.in/raghava/satpdb/catalogs/apd2/
Defensins Knowledgebase	Defensin, antimicrobial	∼360	Exp. Primary	Natural	2007	http://defensins.bii.a-star.edu.sg/

a Data in the database is collected as experimental and/or predicted, which was respectively displayed as the abbreviation ‘Exp. and Pred’. Nonetheless, the primary database is created by manually experimental data and the secondary database is created by using an integration of some other databases. **DBAASP:** Database of Antimicrobial Activity and Structure of Peptides; **LAMP**: Linking antimicrobial peptide; **DRAMP:** Data Repository of Antimicrobial Peptides; **dbAMP:** Database antimicrobial peptides; **InverPep:** Invertebrate peptides; **CAMP:** Collection of antimicrobial peptides; **ADAM:** A Database of Antimicrobial peptides, **APD:** Antimicrobial Peptide Database.

Data bank antimicrobial peptides **(dbAMP)** database contains various information about different types of AMPs in 3044 organisms. Newly, the dbAMP database contains 2 262 antimicrobial proteins and more than 26 440 unique entries, including experimentally verified AMPs and putative AMPs along with their functional activities, which expanded using protein databases of UniProt, NCBI, Protein Data Bank, and eight public AMP databases. In this study, for large-scale detection of AMPs using transcriptome data, all amino acid sequences of AMPs were converted into DNA sequences to create an efficient pipeline using the Docker container for discovering AMPs from Next-Generation Sequencing (NGS) data using the Bowtie2 program. Users can submit large-scale data from NGS reads or peptides identified via MS/MS to the dbAMP. In addition, the system could identify known AMPs with their functional types and predict new AMPs by the constructed model (78).

Database of antimicrobial activity and structure of peptides (DBAASP) contains over 15 700 entries (8000 more than the previous version), including ~14 500 monomers and nearly 400 homo- and hetero-multimers. Of the monomeric AMPs, ~12 000 are synthetic, about 2700 are ribosomally synthesized, and about 170 are non-ribosomally synthesized. DBAASP is freely accessible and contains information about amino acid sequences, chemical structure, target species, the target object of the cell and peptide antimicrobial/hemolytic/cytotoxic activities of peptides. The user can search for peptides based on structural characteristics, complexity type, source, synthesis type (ribosomal, nonribosomal and synthetic) and target species. Importantly, DBAASP provides a prediction tool for the *in silico* design of new AMPs (116).

Linking antimicrobial peptide database (**LAMP**) is an online resource for studying experimentally observed AMPs. LAMP contains natural, synthetic and predicted AMPs and is a useful resource for the discovery and design of AMPs as new antimicrobial agents. LAMP comprises three catalogs of AMPs by data sources: experimental, predicted and patent. AMPs in LAMP are short in length, less than 100 amino acids. Currently, LAMP2, an updated version of LAMP has been created which contains more than 23 250 unique AMP sequences and expands to link 16 public AMP databases. LAMP2 covers ∼ 7, 800 natural AMPs and ∼15, 400 synthetic peptides (117).

Data repository of antimicrobial peptides (**DRAMP**) is another AMPs database and contains useful data about the sequence, structure, antimicrobial activity, physicochemical, patent, clinical and reference information of AMPs. Now, the DRAMP comprises ∼ 22 250 entries, more than 5890 general AMPs (containing natural and synthetic AMPs), ∼ 16 110 patent AMPs and 77 peptides in drug development. DRAMP database contains various information about computational methods obtained from data mining tools and introduces the new design for the development and optimization of AMP-based drugs (5).

A database of invertebrate antimicrobial peptides **(InverPep)** is a database of AMPs belonging to invertebrates. InverPep contains more than 770 experimentally validated AMPs which were manually collected from other databases and scientific literature. Notably, this database contains 33 AMPs that are not reported in other databases. Most AMPs in InverPep are 10 and 50 amino acids in size and positively charged that have 30–50% hydrophobic amino acids. AMP peptides in InverPep have information about their source, physicochemical properties, secondary structure, biological activity and also links to the external literature (118).

Collection of antimicrobial peptides (**CAMP**) is a comprehensive database of sequences, structures and family-specific signatures of prokaryotic and eukaryotic AMPs. Currently, CAMP encompasses more than 8160 sequences, 757 structures, ∼2080 patent AMPs and 114 family-specific signatures of prokaryotic and eukaryotic AMPs. Also, it has provided the tools for sequence alignment, pattern creation and AMP identification (119).

### Specific AMPs databases

Many databases have been created based on certain types, specific sources or certain characteristics of AMPs to search AMPs based on specific classes. Table 2 reviews the current main public AMPs specific databases.

**Table 2.** A description of existing antimicrobial-specific databases

	Specific Statistics					
Database	Type of AMPs	Size	Type of Database	Type of Data	Years	Web site
BaAMPs	Anti-biofilm peptides	∼237	Exp. Primary	Natural BaAMPs	2015	http://baamps.it/
CancerPPD	Anticancer peptides	∼3490	Exp. and Pred. Secondary	Natural, and Predicted ACPs	2015	http://crdd.osdd.net/raghava/cancerppd/
ParaPep	Antiparasitic peptides	∼860	Exp. and Pred. Secondary	Natural, and Predicted APPs	2014	http://webs.iiitd.edu.in/raghava/parapep/peptide.php
YADAMP	Antibacterial peptides	∼2525	Exp. and Pred. Secondary	Natural, and Predicted ABPs	2012	http://yadamp.unisa.it/
DADP	Amphibian peptides	∼2570	Exp. Primary	Natural	2012	http://split4.pmfst.hr/dadp/
THIOBASE	Bacterial thiopeptides	∼100	Exp. and Pred. Secondary	Natural, and Predicted thiopeptides	2012	http://db-mml.sjtu.edu.cn/THIOBASE/
BACTIBASE	Bacteriocins	∼177	Exp. and Pred. Secondary	Natural, and Predicted ABPs	2010	http://bactibase.hammamilab.org/main.php
Cybase	Cyclotides	∼1270	Exp. and Pred. Secondary	Natural, and Predicted AMPs	2008	http://www.cybase.org.au/
Defensins Knowledgebase	Defensins	∼300	Exp. and Pred. Secondary	Natural AMPs	2007	http://defensins.bii.a-star.edu.sg/
peptaibol	Peptaibols	∼317	Exp. Primary	Natural AMPs	2004	http://peptaibol.cryst.bbk.ac.uk/home.shtml

**BaAMPs:** Biofilm-active AMPs database; **ParaPep:** Parasites Peptides; **YADAMP:** Yet another database of antimicrobial peptides; **DADP:** Database of anuran defense peptides; **THIOBASE**: A Database of Thiopeptides Featured in Genetics and Chemistry; **BACTIBASE:** A database dedicated to bacteriocins; **Cybase:** Cyclic protein database; **Defensins:** A manual database on the defensins family of antimicrobial peptides; **Peptaibol:** A database for sequences and structures of naturally occurring peptaibols.

## A brief history of machine learning techniques on AMPs

For the identification of AMPs, high-throughput experimental methods are labor-intensive and time-consuming. Therefore, machine learning (ML) methods and powerful tools to predict AMPs are urgently needed. The advent of high-throughput screening coupled with decades of experimental data allowed for the duration of large annotated datasets (120). In the last 10–15 years, the focus of ML has shifted to an intensely data-driven approach. Significant advancements in computational power and easy-to-use statistical learning tools have made supervised ML a viable strategy for leveraging large datasets for the high-throughput and high-accuracy classification of AMPs. Typical readouts from biophysical assays on AMPs include calculations of minimum inhibitory concentrations, minimum bactericidal concentrations and binding affinities. These quantities, coupled with sequence information about AMPs, allow for the training of various supervised learning models using peptide sequence information as an input. Before this era, methods for *de novo* AMP discovery relied on long-standing bioinformatics methods, including sequence alignment and homology modeling for the prediction of biological activity. Now, the convergence of innovations in ML models, the presence of modern computational tools and the availability of high-quality datasets have enabled the ML-aided design of AMP.

In one of the first applications of ML to AMPs, Lata *et al.* (121) developed a Quantitative Structure–Active Relationship (QSAR) AMP classification tool based on artificial neural network (ANN), support vector machine (SVM) and quantitative matrix models based on unique motifs found in the C- and N-terminal residues of known AMPs. In 2009, Chersakov *et al.* used high-throughput screening methods to train an ANN model on the measured antimicrobial efficacies of thousands of nine-residue peptides to discover potent antimicrobials that were potent against multi-drug-resistant bacteria (122). Fjell *et al.* (2008) published a study using hidden Markov models (HMMs) to screen for AMPs in the bovine genome, which led to the discovery of a previously unknown AMP and confirmed the absence of α-defensins (123). In a similar vein, this group later developed an ANN model in 2009 to screen a larger number of synthetic AMP candidates, characterizing 18 sequences with high antimicrobial efficacy against multi-drug-resistant bacteria (123). Wang *et al.* used a combination of sequence alignment and feature selection methods to design a computational model to more accurately classify AMPs (124). Similarly, Torrent *et al.* (125) trained an eight-descriptor SVM to classify AMPs with 75–90% accuracy while taking into account new factors like peptide aggregation. Maccari *et al.* used random forest (RF) models to design and validate the antimicrobial activity of two natural peptides and one peptide with nonnatural amino acids (126). Giguere *et al.* used a kernel method based on graph theory to train a 100 peptide dataset based on multiple measures of bioactivity to predict novel candidates (127). Most recently, Schneider *et al.* reported the first application of unsupervised–supervised two-step models to classify AMPs. They used self-organizing maps to apply nonlinear dimensionality reduction to the training data, which were then used as an input for a supervised neural network model. Together, these studies highlight a diversity of methods and approaches that have been used to classify and design AMPs with great success (128). Xiao *et al.* designed a two-level classifier to first classify peptide sequences as an AMP, and then sub-classify them into 10 functional AMP categories (129).

## Recent application of machine learning methods for predicting AMPs

Researchers have recently coupled the use of AMP databases as a unique method for AMP prediction with experimental validations to obtain more efficient AMP-based drugs (130). Most of the learning methods discussed in these databases were based on supervised learning and validated datasets of AMPs (34). To this end, ML algorithms including SVM, NN, RF, fuzzy k-nearest neighbor (fuzzy k-NN), HMM, discriminant analysis (DA) and logistic regression (LR) are proposed to identify AMPs (131, 132). These methods had played a key role in the AMP research, nevertheless, they have problems. Models can only tell whether a new sequence is AMP and also, short peptides tend to be harder to find in the database because AMPs are usually only 10–50 amino acids long. Blast search and gene ontology methods are often ineffective in predicting AMPs. Several general databases contain imbalanced datasets of AMPs activities that are not distributed equally (133). The standard ML algorithms often cannot achieve ideal performance when trained on unbalanced AMPs data sets. To solve this problem, existing classifier learning algorithms can be adapted to strengthen learning in the minority class or used to artificially sample the class distribution. Therefore, can achieve strong classifiers via a combination of both approaches. Thus, for classifying AMPs’ functions used a multilabel classification (MLC). During the past 2 decades, the topic of learning from multilabel datasets (MLDs) has been intensively discussed. For example, ML-SMOTE is a new synthetic minority over-sampling technique, designed for processing and identifying AMPs’ functional families based on imbalanced and multilabel datasets (133). A good prediction method combines good unbiased training data, a discriminative feature subset and a suitable learning algorithm. Every computational method for predicting a specific type of AMP based on the sequence information requires the following steps.

### Data gathering

**Figure 4.**
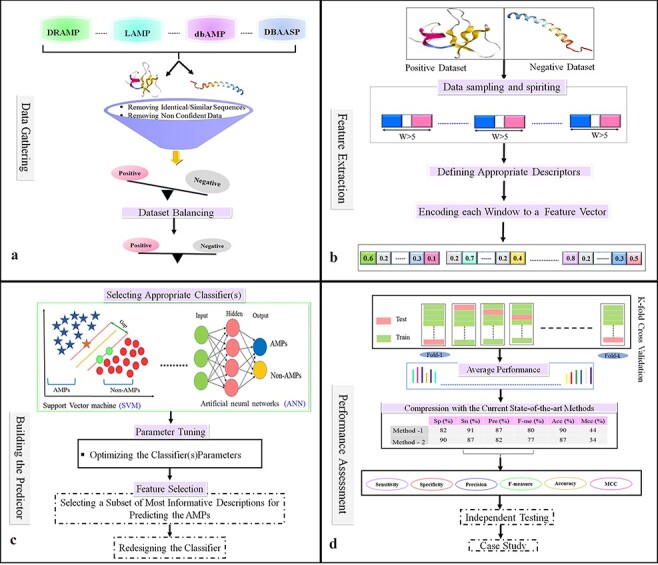
Flowchart of the statistical and machine learning techniques for the detection of AMPs. (a) Collection of data and creation of datasets. (b) Feature selection. (c) Designing training and testing models. (d) Evaluation of model performance.

Select or create a validation benchmark dataset for training and testing the predictor. To assemble a validation dataset, the first step of the AMP prediction method is to collect the data from AMP databases (Figure 4a). It is necessary to include both sequences of peptides with validated AMP activity as positive samples and sequences of peptides with no validated AMP activity as negative samples capable of training a ML algorithm for predicting AMPs. Positive samples are usually collected from the aforementioned databases (such as LAMP or DBAASP). However, the selection of negative samples is the most challenging part of the data collection process and exists main strategies for selecting the negative dataset. A random set of non-AMPs with an equal number of the positive set is selected and thus, are considered as negative samples.

### Filtering and dataset balancing

After assembling both positive and negative datasets, one main goal is reducing homology bias, removing duplicate/inconsistent samples and gaining a more reliable sample set. Depending on the study, this step may be different. Based on the literature, there are three main policies for removing inconsistent/redundant AMPs:

1- Removing identical AMPs and sequence less than five amino acids.

2- Removing similarities within AMPs in the positive and non-AMPs in the negative datasets.

3- Removing similarities between AMPs in the positive and non-AMPs in the negative datasets.

The Cluster Database at high identity with tolerance (CD-HIT) program is used as the major tool to detect similar samples (sequences) and to reduce homology bias and redundancy. However, different studies use different thresholds of identity to consider a pair of AMP sequences to be similar/redundant. In different AMPs prediction studies, this threshold varies from 20% to 100% (134). After filtered datasets there exists an imbalanced dataset and the size of the negative dataset is greater than the size of the positive dataset. These imbalanced datasets can create biases in the learning phase when a learning method is not a sufficient algorithm. Consequently, to ensure dataset balancing, a subset of the negative dataset equal to that of the positive dataset will be chosen (Figure 4a).

### Feature extraction

Selecting suitable algorithms to learn patterns and distinguish AMPs from other sequences, feature generation, extraction, engineering and selection became essential aspects of finding good representative features or informative features that could capture AMP patterns and increase prediction accuracy. To select features that differentiate AMPs from non-AMPs, a feature selection method was almost used. Thus, the positive or negative samples (peptide sequences), according to their biological properties, are coded into numerical feature vectors that are used for learning the proposed model (135). Then, each peptide is encoded as a numerical feature vector based on suitable biological features, such as physicochemical properties, sequence composition, and structural features (Figure 4b).

### Training the predictors

Several learning algorithms will be used to compare the effectiveness of the variable features selected. This process must take place before the final prediction model is built. As part of feature selection, a subset of the most informative/discriminative features is selected and used to train the classifier. A good classifier can be chosen according to the performance of various classification methods. As a result of parameter optimization, the algorithm is trained on a subset of the assembled dataset (training dataset), and then it can be evaluated and compared against the current state-of-the-art methods (Figure 4c).

### Performance assessment

There are two methods for evaluating models: Independent test (Train-Test) and K-fold cross-validation. In the independent test method, a dataset is divided into two sets, a Train-Validation dataset and a Test dataset. Afterward, the Train-validation set also splits into two subsets: the train set and the validation set. A training set is used to train models, and a test set is used to evaluate the models and select the best model according to performance via evaluation of the test set. On the other hand, the validation set evaluation results differ from the train set evaluation results, it shows the model has been overfitted to the train set. Lastly, the test set should be reported, and there should not be a huge difference between the validation and test sets.

K-fold cross-validation is a standard procedure for assessing the performance of a given classifier and is used to evaluate ML models on a limited data sample. In this process, the available dataset is randomly divided into *k* subsets without any overlap. One of the subsets is used as a test set dataset, and the other as training for assessing the predictor. Each subset is used exactly once as the test set, and the process is repeated *k* times. Finally, the average performance for all *k* test sets is considered (Figure 4d). Cross-validation with k-fold is typically used when there is only a limited amount of Train-Validation data, and the Train-Validation method is mostly used when massive amounts of data are accessible. K-fold is used in most classified AMPs methods.

## Model evaluation criteria

Assessing the performance of the AMPs prediction methods based on the four basic parameters which are explained as follows:

1-‘True positive’ (TP): the experimentally validated AMPs that have been correctly predicted by the prediction method.

2-‘True negative’ (TN): the non- AMPs sites that have been correctly predicted.

3-‘False positive’ (FP): the non- AMPs that have been incorrectly predicted as AMPs.

4-‘False negative’ (FN): the experimentally validated AMPs that have been incorrectly predicted non- AMPs.

The classification performance is often evaluated by accuracy, sensitivity (Recall), specificity, precision, F-measure and Matthews correlation coefficient (MCC). All performance criteria for AMPs prediction are shown in Equations (1–6). Alongwith the abovementioned measures which are known as threshold-dependent measures, ROC (receiver operative characteristic) and AUC (area under the ROC-curve) are two main threshold independent evaluation measures (136, 137). The most important assessment measures based on the abovementioned parameters have been described in the following section.

**Sensitivity:** Sensitivity or recall indicates the percentage of samples that have been predicted correctly.

(1) $$Sensitivity = {\ }{{TP} \over {TP + FN}}{\ } \times 100$$

**Specificity:** Specificity shows the percentage of negative samples that have been predicted correctly as negative samples.

(2) $$Specificity = {\ }{{TN} \over {TN + FP}} \times 100$$

**Accuracy:** Accuracy is a ratio between the correctly classified data points to the total number of samples (138).

(3) $$Accuracy = {\ }{{TP + TN} \over {TP + FP + TN + FN}}{\ } \times 100$$

**Precision:** Precision or positive prediction value (PPV) is shown as the ratio of the number of correctly predicted positive samples to the total number of positive samples (138).

(4) $$\textrm{Precision} = {{TP} \over {TP + FP}} \times 100$$

**F-measure:** This metric represents the harmonic mean of recall and precision, and is calculated as:

(5) $$\textrm{F-measure} = {{2TP} \over {2TP + FP + FN}} \times 100$$

**Matthews Correlation Coefficient (MCC):** MCC shows the correlation between true and predicted labels (139).

(6) $$MCC =\,& {{TP \times TN - FP \times FN} \over {\sqrt {\left( {T{\rm{P}} + TN} \right)\left( {TP + FN} \right)\left( {TN + FP} \right)\left( {TN + FN} \right)} }}\nonumber\\& \times 100$$

## Tools for AMPs prediction

Considering the high cost and labor-intensive experimental identification of AMPs, many computational methods have been proposed for the prediction of AMPs and their functional types which can be useful to design new and more effective antimicrobial agents. The attention to ML has been converted to a strongly data-driven approach. As a result, with development in computational methods and tools, supervised learning can be considered as a suitable strategy for leveraging large datasets for the high-throughput and high-accuracy classification of AMPs (34). Studies have shown differences in amino acid composition (AAC), the physicochemical property, sequence order and the pattern of terminal residues in AMPs that can affect AMP prediction. Furthermore, it has been reported that the net charge, isoelectric point, composition and tendency for the secondary structure are different in the AMPs, like antibacterial, antifungal and antiviral activities, and as a result, these features should be used for learning algorithms for AMPs prediction (140). Many of these methods have been implemented as publicly accessible tools. However, there is still a lack of efficient prediction models to identify potential peptides with specific activities. An overview of existing predictive tools supporting AMP studies is presented in Table 3. Indeed an explanation of the newest comprehensive tool was provided.

**Table 3.** Online AMPs prediction tools

Acronym	Features	Method	Validation Method	Years	URL
Ensemble-AMPPred	517 features and a hybrid feature Amino acid composition, pseudo amino acid composition (PseAAC) in parallel and series correlation, and the details of the secondary structure conformation, composition–transition–distribution (CTD), various physical-chemical properties, antimicrobial propensity scale, and the percentage of different conformations in the peptide sequence.	Ensemble learning method	10-fold CV, Independent test	2021	http://ncrna-pred.com/Hybrid_AMPPred.htm
DBAASP	Physicochemical characteristics of peptides: normalized hydrophobic moment, normalized hydrophobicity, net charge, isoelectric point, penetration depth, tilt angle, disordered conformation propensity, linear moment, and propensity for *in vitro* aggregation	Cutoff discriminator	5-fold CV, Independent test	2021	http://dbaasp.org/home
Deep-AmPEP30	AMPs in sequences Pseudo K-tuple RAAC	Deep Learning	10-fold CV, Independent test	2020	http://cbbio.online/AxPEP/
AntiCP	ACPs in sequences Amino acid composition, dipeptide composition, terminus composition, binary profile, and hybrid features	Support Vector Machine	5-fold CV, Independent dataset	2020	https://webs.iiitd.edu.in/raghava/anticp2/
AmpGram	AMPs in sequences	Random Forest	5-fold CV, Independent dataset	2020	http://biongram.biotech.uni.wroc.pl/AmpGram/
AMPScanner	Numerical matrix from deep neural network (DNN)	Deep Learning	10-fold CV, Independent dataset	2018	https://www.dveltri.com/ascan/
AntiMPmod	AMPs in structures	Support Vector Machine	5-fold CV Independent dataset	2018	https://webs.iiitd.edu.in/raghava/antimpmod/
PscAAC	AFPs in sequences and structures	Support Vector Machine	10-fold CV, Independent dataset	2018	http://www.csbio.sjtu.edu.cn/bioinf/PseAAC/
MLAMP	PseAAC with the gray model (GM)	ML-SMOTE	Independent dataset	2016	http://www.jci-bioinfo.cn/MLAMP
CAMPR3	Sequence composition, physicochemical properties, and structural characteristics of amino acids	Support Vector Machine, Random Forests, and	10-fold CV, Independent dataset	2016	http://www.camp.bicnirrh.res.in/prediction.php
CPPpred	cell-penetrating peptides in sequences	N-to-1 neural networks	5-fold CV, Independent dataset	2013	http://bioware.ucd.ie/∼compass/biowareweb/Server_pages/cpppred.php
iAMP-2 L	Pseudo amino acid composition (PseAAC) incorporating five physicochemical properties	fuzzy K-nearest neighbor	Independent dataset	2013	http://www.jci-bioinfo.cn/iAMP-2L
PeptideLocator	Bioactive peptides in sequences	Bidirectional Recursive Neural Networks	5-fold CV, Independent dataset	2013	http://bioware.ucd.ie/∼compass/biowareweb/
BAGEL3	Bacteriocins in DNA sequences	BLAST ORFs prediction tools	—-	2013	http://bagel.molgenrug.nl/
CS-AMPPred	cysteine-stabilized AMPs in sequences	Support Vector Machine	5-fold CV	2012	http://sourceforge.net/projects/csamppred/
AMPA	Antimicrobial index based on IC50 value	Antimicrobial propensity scale threshold	——	2011	http://tcoffee.crg.cat/apps/ampa/guide.html

### Ensemble-AMPPred

In this work, several well-known single and ensemble (ML) approaches have been explored and evaluated based on balanced training datasets and two large testing datasets. They have demonstrated that the developed program with various predictive models has high performance in differentiating between AMPs and non-AMPs. The present work used a benchmark AMP dataset consisting of 920 AMPs and 920 non-AMPs in testing existing AMP prediction programs and detected the false predictive answers of each program are different. The results suggest that, due to the use of different models and features exist unpredictable answers that have a different distribution. Because of these limitations, each program should consider improvements, including minimizing false positives (FPs) and increasing predictive accuracy. Due to the mentioned points, they considered using of integrating different learning models using ensemble learning techniques. In ensemble learning techniques, using multiple predictors and ensemble methods for incorporating individual classification models (bagging and boosting) leads to a decrease in FPs and increasing prediction accuracy. In this study, AMP prediction models were developed using ensemble methods based on five different algorithms, as well as comparing four different single models (135).

Data from 15 public available bioactive peptide databases were collected by Ensembl-AMPPred, and peptides with sequences shorter than 10 amino acids were removed. CD-HIT program for reducing data redundancy with a threshold of 0.9 (90% sequence similarity) was used. Finally, 13 434 peptides were considered as positive sequence data. Using Uniprot, negative data was collected on proteins without antimicrobial activity and a secretory signal peptide position. The basic local alignment search tool (BLAST) was used to remove AMP matches and peptide sequences with lengths <10 amino acids. Furthermore, peptide sequences that had an identity greater than 25% were removed using the CD-HIT program. As a result, 37 595 peptides are considered negative. Also, the similarity between the positive and negative datasets was removed, and as a result, the sequence similarity between the training and testing datasets was calculated as 47.29%. Lastly, the training data includes 1800 sequences of peptides from the AMP dataset, and 1800 sequences from the non-AMP dataset were prepared (135). A dataset consisting of 517 peptide features was then extracted and to filter this feature vector a logistic regression was applied to create a hybrid feature vector using four preselected single features based on an equation for logistic regression. Finally, a performance comparison of eight single predictive models and five ensemble models was done and prediction accuracy was evaluated using 10-fold cross-validation. Although ensemble models have better performance than single models, these four models (RF, NN, SVM and LDA) showed the highest performance among single models. Ensemble models have better performance than single models. Nevertheless, four models (RF, NN, SVM and LDA) showed the highest performance among single models. Thus, Ensemble-AMPPred is an AMP predictor which able to high performance in differentiating between AMPs and non-AMPs in comparing other available methods (135).

## Conclusion

The AMPs constitute an important component of innate immunity and are effective against disease-causing pathogens. Multidrug-resistant bacteria (superbugs) are on the rise, making AMPs an important alternative to traditional antibiotics. However, the identification of AMPs through lab experiments is still expensive and time-consuming, and, most importantly, ineffective due to the staggering number of genomes being sequenced today. Therefore, the development of an efficient computational tool is essential to identify the best candidate AMPs with high accuracy before the *in vitro* experiments. Thus, the bioinformatics resources and the usage of computational tools to analyze AMPs data and their functional outcomes across species are crucial. There would be a significant interest in the development of computational methods for the reliable prediction of AMPs. Recently, many advanced computational methods and tools have been developed to predict AMPs, and many of them are publicly available. Therefore, in the near future, research in databases could be a key step in developing a typical new antimicrobial agent .
